# Numerical modeling of the abdominal wall biomechanics and experimental analysis for model validation

**DOI:** 10.3389/fbioe.2024.1472509

**Published:** 2024-09-27

**Authors:** Silvia Spadoni, Silvia Todros, Piero G. Pavan

**Affiliations:** ^1^ Department of Industrial Engineering, University of Padova, Padova, Italy; ^2^ Fondazione Istituto di Ricerca Pediatrica Città della Speranza, Padova, Italy

**Keywords:** abdominal wall, numerical modeling, muscle contraction, intra-abdominal pressure, *in vivo* experiments

## Abstract

The evaluation of the biomechanics of the abdominal wall is particularly important to understand the onset of pathological conditions related to weakening and injury of the abdominal muscles. A better understanding of the biomechanics of the abdominal wall could be a breakthrough in the development of new therapeutic approaches. For this purpose, several studies in the literature propose finite element models of the human abdomen, based on the geometry of the abdominal wall from medical images and on constitutive formulations describing the mechanical behavior of fascial and muscular tissues. The biomechanics of the abdominal wall depends on the passive mechanical properties of fascial and muscle tissue, on the activation of abdominal muscles, and on the variable intra-abdominal pressure. To assess the quantitative contribution of these features to the development and validation of reliable numerical models, experimental data are fundamental. This work presents a review of the state of the art of numerical models developed to investigate abdominal wall biomechanics. Different experimental techniques, which can provide data for model validation, are also presented. These include electromyography, ultrasound imaging, intraabdominal pressure measurements, abdominal surface deformation, and stiffness/compliance measurements.

## 1 Introduction

Understanding the mechanical behavior of the abdominal wall can help in the investigation of its healthy and pathological conditions. Despite many efforts made over the years, the biomechanics of the abdominal wall is still not fully understood from a quantitative point of view, due to the complex geometry and mechanics of the anatomical region, the lack of extensive experimental datasets, the high costs of clinical trials, and related ethical aspects. Increasing knowledge on the biomechanics of the abdomen can be useful, in particular, to address rational design and use of surgical meshes for hernia repair (Deeken and Lake, 2017).

*In silico* analysis through the Finite Element Method (FEM) has shown the ability to provide a deeper understanding of the mechanical properties of biological tissues, lowering costs and time with respect to an experimental or clinical approach. FEM-based models can consider the geometric and material properties of the anatomical structures and simulate the interactions between muscle fibers, fascial tissue, and other involved tissues. By applying external loads or constraints to the model, it is possible to simulate a wide range of scenarios and analyze the resulting deformations, stresses, and strains within each tissue.

A mandatory aspect of this process is the validation of FEM models, which consists in verifying that the obtained numerical results are feasible. Generally, this can be achieved by replicating specific experimental conditions by means of numerical analysis, and comparing numerical outcomes and experimental data. Alternatively, or in combination, the results can be compared to the outcomes from similar–but already validated–models. Once validated, the models can be adopted to enlarge the numerical analysis to a broader range of conditions. To assess the required accuracy of FEM models, it should be necessary to first evaluate the variability of biological data, including anthropometric characteristics of the anatomical regions and mechanical properties of the constituent tissues, due to age, sex, BMI or presence of pathologies. Some considerations about the importance of the validation process are proposed in the Discussion section.

FEM models can be also personalized to individual anatomical variations, allowing patient-specific simulations. This capability is particularly relevant in clinical applications, such as in the surgical planning or in the design of patient-specific prosthetics. By incorporating medical imaging data, such as Computed Tomography (CT) or Magnetic Resonance Imaging (MRI), FEM models can potentially be tailored to accurately represent the unique anatomy of an individual patient.

Advances in imaging technology, computational power, and material modeling techniques have significantly improved the precision and predictive capabilities of numerical models. Therefore, computational methods have the potential to provide information on the mechanical behavior of abdominal muscles under different loading conditions, contributing to a better understanding of their function, the development of improved rehabilitation strategies, and the design of innovative medical devices.

Investigating the biomechanics of the abdomen is particularly important in the case of pathologies. Among all pathologies, those that affect the abdominal wall, such as hernia, show a prevalence of 1.7% for all ages and 4% for people over 45 years (Jenkins and O’Dwyer, 2008). The most common surgical technique for hernia repair is the laparoscopic approach with a surgical mesh (Heniford et al., 2003; Colavita et al., 2013), where the selection of the most appropriate prosthesis is mainly based on the surgeon’s experience (Mudge and Hughes, 1985). As shown in several follow-ups, an improper solution can cause discomfort and postoperative pain for the patient; therefore, choosing the most suitable mesh is crucial. This selection may be supported by FEM analysis, which could provide information on the effects of different mesh configurations and sizes, considering the complexity of the surrounding anatomical region.

To study the biomechanics of the abdominal wall, it is fundamental to account for the activation of the abdominal muscles and to understand its correlation with intra-abdominal pressure (IAP) during different physiological functions and motor tasks (Bjerkefors et al., 2010). In this context, the introduction of minimally invasive instruments and imaging methods has revolutionized clinical practice (Meier et al., 2001); in particular, electromyography (EMG), ultrasound (US) imaging, IAP measurements, and the evaluation of abdominal deformation and compliance represent fundamental tools to evaluate abdominal behavior *in vivo*. Integration of EMG, US imaging, deformation, and IAP measurements has significantly improved our understanding of abdominal muscle function and its role in various contexts, such as sports performance, injury prevention, and rehabilitation. These technologies allow researchers and clinicians to assess muscle activation patterns, visualize muscle structure, and measure muscle deformation and mechanical properties during different tasks. This knowledge is essential to design effective numerical models, identify muscle imbalances or dysfunctions, and develop targeted rehabilitation strategies. Continuous progress in these technologies will undoubtedly contribute to further advances in understanding the function of abdominal muscles and their influence on human performance and health.

The aim of this work is to review the different FEM models developed in the literature, as well as several measurement techniques used to develop and validate these models. For this purpose, the anatomy of the human abdominal wall is described first, as a basis for defining the geometry of FEM models. The mechanical properties of abdominal tissues are presented, according to experimental tests available in the literature, on the abdominal wall of human subjects. These data are useful for the development of constitutive models capable of describing the behavior of abdominal tissue in FEM models. Then, computational models of the abdominal wall are presented, including mostly passive models and also considering a few cases with active muscular behavior. Lastly, *in vivo* measurements in human subjects, such as EMG, US imaging, IAP measurements, abdominal surface deformation, and stiffness measurements, are collected. This overview allows evaluating the limited availability of *in vivo* data for the validation of FEM models and highlighting possible gaps to be filled for a deeper understanding of abdominal biomechanics.

## 2 Abdominal wall anatomy

The abdominal wall includes seven layers: skin, subcutaneous tissue, superficial fascia, deep fascia, muscle, extraperitoneal fascia, and peritoneum.

According to Lancerotto et al. (2011), there are three layers under the dermis in the subcutaneous tissue of the anterior abdominal region: a superficial adipose layer, a membranous layer and a deep adipose layer. These layers cover the deep fascia that encloses the muscles of the abdominal wall.

The abdominal muscles include the External Oblique (EO), Internal Oblique (IO), Transversus Abdominis (TA), and Rectus Abdominis (RA), which are interdigitated with each other and ensure core strength (Flynn and Vickerton, 2022). In the anterior part of the abdominal wall, each flat muscle forms an aponeurosis (AP) that covers the RA muscle. The APs of all flat muscles are linked in the midline, forming Linea Alba (LA), a fibrous structure that extends from the xiphoid process of the sternum to the pubic symphysis. The RA runs vertically down the front of the abdomen and is responsible for flexing the spine and creating spinal stability. The IO and EO are located on the sides of the abdomen and help in rotating and bending the trunk. The role of the abdominal wall is essential not only in protecting the visceral structures, but also in stabilizing the trunk and distributing loads (Grevious et al., 2006).

**FIGURE 1:**
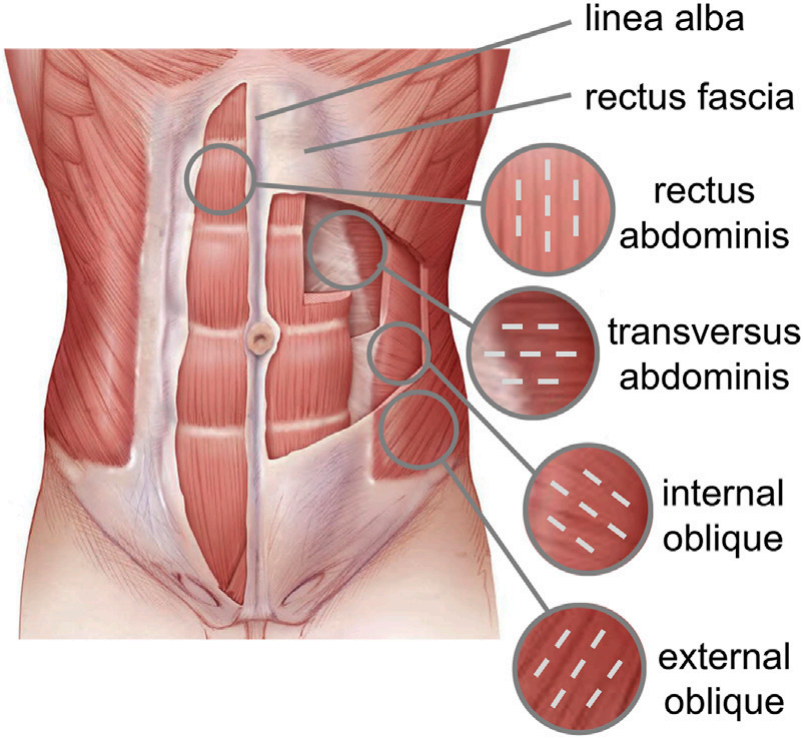
Abdominal wall anatomy with different fiber orientations highlighted with dotted lines.

The spatial orientation of the muscle fibers is different for each abdominal muscle (Figure 1). The EO fibers diffuse caudally to the iliac crest and inguinal ligament and medially to the LA, the IO fibers emerge from the inguinal ligament and iliac crest and are inserted into the anterolateral surface of the cartilages of the last three ribs and into the LA perpendicular to the EO fibers. The TA fibers extend circumferentially in a downward direction, while the RA muscle fibers are parallel to the LA.

An interesting analysis of the architecture of the abdominal wall muscles can be found in Brown et al. (2011), where a correlation between the length of the sarcomere and the biomechanical functions of each muscle is proposed. Based on cadaveric data, RA shows the lowest Physiological Cross Area (PCA), associated with the highest sarcomere length (3.29 ± 0.07 μm) among all abdominal muscles; this can be correlated with the generation of the smallest isometric force. Differently, IO is characterized by the largest PCA and the smallest sarcomere length (2.61 ± 0.06 μm), thus being able to generate the highest contraction force among all abdominal muscles, but with a small range of motion.

Abdominal muscles are enveloped by a thin epimysial fascia, allowing the various muscular layers to glide. In particular, the fascial layers surrounding the RA are divided into Anterior and Posterior Rectus Sheath (ARS and PRS, respectively). In the ARS, oblique bundles of collagen fibril are interlaced with each other, whereas the PRS consists predominantly of transverse fibril bundles (Axer et al., 2001). This structural conformation is considered responsible for the mechanical anisotropy of both ARS and PRS (Astruc et al., 2018). Proximally, the transversalis fascia (TF) separates the anterior abdominal wall from the extraperitoneal fat, while posteriorly, it is continuous with the thoracolumbar fascia. Understanding the structure and biomechanical role of abdominal fasciae is relevant from a surgical point of view, for example, in the evaluation of the choice of direction of laparotomy incision, and in the analysis of the overall biomechanics of the abdominal wall.

## 3 Review methodology

The literature research was conducted using the English-language databases PubMed, Web of Science and Elsevier ScienceDirect. Keywords and inclusion criteria adopted were different for each of the topics described and are therefore specified in the following.

The review of the numerical models of abdominal wall biomechanics is systematic and covers–to the best of the knowledge of the authors–the relevant works published on the topic. The sections dedicated to the experimental testing on abdominal wall are focused on collecting those elements that can be relevant for the development and validation of numerical models of the abdominal wall. Search was not restricted to specific geographic regions and also references present in the articles were included.

### 3.1 Mechanical characterization of abdominal wall tissues

The following combination of keywords was considered: (“tensile test” OR “mechanical test”) AND (“human abdominal wall” OR “human abdominal muscle” OR “human linea alba” OR “human abdominal fascia”). The study inclusion criteria were as follows: experimental test need to be on human abdominal tissues, experimental protocols needed to be described, quantitative results about the stress-strain behavior had to be reported. Eight articles were selected.

### 3.2 Numerical modeling of the human abdominal wall

The following combination of keywords was considered: (“finite element model” OR “finite element method” OR “FEM”) AND (“human abdominal wall” OR “human abdomen” OR “abdominal wall contraction” OR “abdominal muscles” OR “abdominal hernia”). The study inclusion criteria were as follows: the FEM models needed to include the human abdominal wall, detailed methodology for the development of the abdomen FEM models needed to be described, the different abdominal tissues needed to be presented (studies with rough monolayer models were discarded), FEM models needed to describe the mechanical behavior of the human abdominal wall in passive and/or active condition of the muscles. Thirteen articles were selected.

### 3.3 Electromyography

The following combination of keywords was considered: (“electromyography” OR “EMG”) AND (“human abdominal wall” OR “human abdominal tissue” OR “human abdominal muscle” OR “abdominal exercise”). The study inclusion criteria were as follows: experimental protocols needed to be described, results about one or more human abdominal muscle had to be presented, results had to include quantitative description of muscle response, results had to describe muscle activation during different motor tasks. Twenty-seven articles were selected. Among these, seven articles in which the experimental testing included also simultaneous ultrasound imaging were presented in a separate section (Paragraph 6.3).

### 3.4 Ultrasound imaging

The following combination of keywords was considered: “ultrasound imaging” AND (“human abdominal wall” OR “human abdominal muscle” OR “abdominal contraction”). The study inclusion criteria were as follows: experimental protocols had to be described, results about one or more human abdominal tissues had to be presented, results had to include quantitative description of muscle behavior, results needed to describe muscle activation during different motor tasks and/or change in muscle thickness. Thirty-one articles were selected. Among these, seven articles in which the experimental testing included also simultaneous electromyography were presented in a separate section (Paragraph 6.3).

### 3.5 Intra-abdominal pressure measurement

The following combination of keywords was considered: (“intraabdominal pressure” OR “intra-abdominal pressure” OR “IAP”) AND (“human abdominal wall” OR “motor task”). The study inclusion criteria were as follows: experimental protocols needed to be described, results needed to include measurements of the intraabdominal pressure variation related to specific activities or motor tasks in human subjects. Fourteen articles were selected.

### 3.6 Stiffness measurements

The following combination of keywords was considered: (“stiffness” OR “compliance”) AND “human abdominal wall”. The study inclusion criteria were as follows: experimental protocols needed to be described, results needed to include measurements of the human abdominal stiffness, experimental tests needed to include *in vivo* studies. Five articles were selected.

### 3.7 Surface deformation measurements

The following combination of keywords was considered: (“surface” OR “deformation” OR “optical measurement”) AND “human abdominal wall”. The study inclusion criteria were as follows: experimental protocols needed to be described, results needed to include measurements of deformation on human living subjects. Six articles were selected.

## 4 Mechanical characterization of abdominal wall tissues

A limited number of studies in the literature investigate the mechanical properties of the human abdominal wall, mainly due to the limited availability of human samples and to the concurrent issues in tissue preservation. Furthermore, this approach can consider only the passive behavior of the tissues and cannot include the evaluation of the active behavior of abdominal muscles.

In general, most of the experimental studies in the literature focus on specific layers of the abdominal wall, which are dissected and isolated from other abdominal structures.

Several works investigate the mechanical behavior of LA. Gräβel et al. (2005) evaluate the compliance of human LA in longitudinal and transverse directions to assess anisotropy in a high number of subjects (15 female and 16 male). They find that LA compliance is about two times higher in the longitudinal than in the transverse direction; moreover, some comparisons between the mechanical properties of LA in men and women are proposed. Hollinsky and Sandberg, (2007) measure the ultimate tensile stress for human LA of 66 cadaveric subjects with mean age of 77 (range: 17–94 years) in the transverse and longitudinal direction. In the epigastric region, they find mean values of 4.5 ± 1.0 MPa and 10.0 ± 3.4 MPa in the longitudinal and transverse direction, respectively; in the hypogastric region, they estimate mean values of 4.1 ± 2.5 MPa and 8.4 ± 3.1 MPa in the longitudinal and transverse direction, respectively. Förstemann et al. (2011) carry out uniaxial tensile tests up to failure on samples obtained from 6 donors to measure the ultimate membrane force in the transversal and longitudinal directions. The mean values reported for the ultimate membrane force are 7.5 N/mm and 1.1 N/mm in the transverse and longitudinal directions, respectively, showing a high strength ratio. Levillain et al. (2016) perform uniaxial tensile tests, comparing human and porcine LA, and evaluating the correlation of mechanical properties with the distribution of elastin and collagen fibers. According to their results, human and porcine LA show similar microstructure and nonlinear anisotropic mechanical behavior; however, porcine LA was approximately 1.5 times stiffer than human LA.

Therefore, according to these works, LA is characterized by anisotropic mechanical behavior, showing higher stiffness and strength in the transverse direction compared to those in the longitudinal direction. A direct comparison among the proposed data is complicated by differences in mechanical test protocols and measurements.

Other studies are dedicated to the analysis of the mechanical behavior of human ARS up to failure under uniaxial loading conditions. Hollinsky and Sandberg, (2007) present experimental data from 66 cadaveric subjects with a mean age of 77 (range: 17–94 years) showing an ultimate tensile stress equal to 8.1 ± 2.1 MPa and 3.4 ± 1.6 MPa for the ARS in the epigastric region in the transverse and longitudinal directions, respectively, and equal to 8.5 ± 2.5 MPa and 3.4 ± 2.0 MPa for the ARS in the hypogastric region in the transverse and longitudinal directions, respectively.

Martins et al. (2012) perform uniaxial tensile tests on samples from 12 female donors, finding a Young’s modulus of 30.3 ± 10.5 MPa and 10.1 ± 5.3 MPa in the longitudinal and transverse directions, respectively. Ben Abdelounis et al. (2013) evaluate the mechanical response of three human ARS using two loading rates, corresponding to quasi-static (0.01 s^−1^) and almost-instantaneous (50 s^−1^) strain rates. The mean values of the Young’s modulus, equal to 5.6 MPa and 14 MPa, are found for the quasi-static and almost-instantaneous test, respectively.

As mentioned above, only a few contributions give an overall view of the mechanical properties of the human abdominal wall including the different muscles. Cardoso, (2012) evaluates 119 samples of FT, RA, TA, EO, and IO from 12 cadavers. All muscles are tested in uniaxial tensile mode in the direction of the fibers; characteristic parameters are obtained, among which the secant modulus $E_{s}$ , the tangent modulus $E_{t}$ , the maximum tensile stress $\sigma_{\max}$ , and the corresponding ultimate stretch $\lambda_{U}.$ This study also explores the influence of age, sex and body mass index (BMI) on the mechanical properties of the abdominal tissues. The parameters obtained in the study are reported in Table 1.

**TABLE 1:** Mean parameters obtained by Cardoso, 2012 from uniaxial experimental tests: secant modulus $E_{s}$ , tangent modulus $E_{t}$ , maximum tensile stress $\sigma_{\max}$ , and corresponding ultimate stretch $\lambda_{U}.$

	$E_{s}\;\left(\text{MPa}\right)$	$E_{t}\;\left(\text{MPa}\right)$	$\sigma_{\max\;}\left(\text{MPa}\right)$	$\lambda_{U}$
FT	3.54	9.06	2.86	1.56
EO	0.33	1.00	0.57	1.98
IO	0.26	0.65	0.39	1.94
RA	0.33	0.52	0.23	1.60
TA	0.31	1.03	0.73	2.19

In a more recent contribution Kriener et al. (2023) characterize the tensile properties of each layer of the human abdominal wall from 15 cadaveric subjects, comparing samples from fresh-never-frozen (FNF) and fresh-frozen (FF) cadavers. They collect a total of 232 samples in longitudinal and transverse directions for ARS and PRS, peritoneum, LA, RA, EO, IO, and TA. Samples are tested at a strain rate varying between 0.01 s^−1^ and 0.006 s^−1^ and the tangent elastic modulus *E* of each tissue layer is evaluated in the almost-linear portion of the stress-strain curve, following the toe region. The values obtained are reported in Table 2. According to these data, FF tissues are generally stiffer than FNF tissues, except in the case of PRS, peritoneum, and TA.

**TABLE 2:** Tangent elastic modulus *E* (median and interquartile range) of abdominal wall tissues from FNF and FF samples (Kriener et al., 2023).

	*E* (MPa)	
	FNF	FF
ARS	6.02 (10.14)	8.29 (15.76)
PRS	14.32 (57.62)	7.31 (67.69)
LA	2.67 (6.87)	3.92 (3.80)
peritoneum	6.79 (6.09)	4.42 (14.15)
RA	0.19^a^	0.30 (0.88)
EO	0.24 (0.37)	0.57 (1.27)
IO	0.42 (0.068)	0.58 (1.11)
TA	2.75 (5.42)	0.81 (2.44)

^a^ Sample size is three.

As reported above, the experimental data coming from uniaxial tensile tests of human abdominal tissues are consistent, even if with high variability. However, there is a lack of data in the biaxial tensile mode, which would be useful to better understand the biomechanics of the abdominal wall under physiological loading conditions. In fact, the abdominal wall experiences multidirectional loads and deformations during daily activities, such as bending, twisting, and stretching.

Another lack in the literature is the study of the active behavior of single muscle fibers or bundles derived from human abdominal wall muscles. These studies would allow us to obtain information on the maximum isometric tension, which is a necessary parameter to assess the contractile capability of muscular tissue.

## 5 Numerical modeling of the human abdominal wall

Numerical modeling, generally based on the FEM approach, can be useful for obtaining quantitative information on the biomechanics of the abdominal wall and to assess different healthy and pathological conditions. In fact, a simple approach based on experimental analyses does not allow the understanding of a complex biomechanical scenario that involves muscle contraction, IAP variation, non-linear mechanical response of the tissues, and a complex anatomy.

A large part of the literature in this field is focused on the passive mechanical response of the human abdominal wall. Hernández-Gascón et al. (2014) develop a FEM model of the abdominal wall with a simplified geometry, aiming at evaluating the performance of hernia meshes in their interaction with the surrounding tissues. In particular, the focus is on numerical aspects concerning the constitutive modeling of surgical meshes, by using refined or more simple approaches. The abdomen is modeled as an extruded ellipse with a size compatible with a male abdomen, and the abdominal wall is considered as a single muscular layer with a thickness of 15 mm. This layer is described adopting a hyperelastic anisotropic constitutive model with parameters based on experimental data previously acquired from an animal model (Hernández et al., 2011). Numerical analyses simulate the effects of an intra-abdominal pressure (IAP) of 171 mmHg (corresponding to jumping) on a herniated wall repaired with a surgical mesh. The results of this work focus on the comparison of different surgical meshes and modeling methods, even though the geometry of the abdomen is simplified. In fact, the analysis of the biomechanics of the abdominal wall considering the effective geometry is beyond the scope of the work.

A more detailed FEM model of the human abdomen is proposed by Hernández-Gascón et al. (2013a) to study the passive mechanical response related to physiological tasks. The geometry of the model is extracted from MRI data from a healthy 38-year-old man. The LA, RA, AP and lateral muscles are identified by manual segmentation and the lateral muscles are described as single layer due to the difficulty in recognizing IO, EO, and TA from medical images. The model includes TF, ARS, PRS, diaphragm, and pelvis, while skin and fat are not included due to their negligible stiffness. Abdominal muscles–in their passive behavior–and aponeuroses are modeled as fiber-reinforced hyperelastic materials, while the diaphragm and pelvis are described assuming a neo-Hookean hyperelastic formulation. The constitutive parameters are set on the basis of experimental data from both human subjects (when available) and animal models. The basal stress state of the abdominal muscles in the geometrical configuration corresponding to MRI data of the subject in supine position is obtained by an optimization procedure. This model is used to simulate the mechanical response of the abdominal wall to the IAP induced by physiological loads, assessing the deformation profile of the abdomen in the craniocaudal and mediolateral directions related to the level of IAP.

This model is used as a basis to simulate the occurrence of hernia and the interaction between the abdominal wall and different types of meshes after surgical repair (Hernández-Gascón et al., 2013b), applying an IAP corresponding to different motor tasks. Numerical results show that the overall mechanical response, as well as the stress acting on the prostheses, are significantly affected by the levels of surgical mesh anisotropy and stiffness.

A similar approach is used by Pachera et al. (2016) to develop a model describing the passive behavior of an abdominal wall in physiological conditions and the mechanical response under different IAP corresponding to daily life activities. The geometry of the model is reconstructed from MRI data of a healthy male subject and includes LA, RA, a single structure that resembles the lateral muscles (TA, IO, and EO), AP and all fascial tissues. The model is simplified by assuming a symmetry with respect to the sagittal plane. Based on data from tensile tests on human abdominal tissues (Förstemann et al., 2011; Cardoso, 2012; Ben Abdelounis et al., 2013), fiber-reinforced and almost-incompressible hyperelastic constitutive models are assumed to describe the mechanical response of the different tissues. Numerical results show the deformed configuration of the abdominal wall at different IAPs and are compared to experimental data acquired on human cadavers (Konerding et al., 2011) and living subjects (Song et al., 2006), confirming the reliability of the model. The previous model is then adopted to numerically simulate the occurrence of a hernia and surgical repair (Todros et al., 2018), focusing on the consequent changes in compliance of the abdominal wall in passive condition.

**FIGURE 2:**
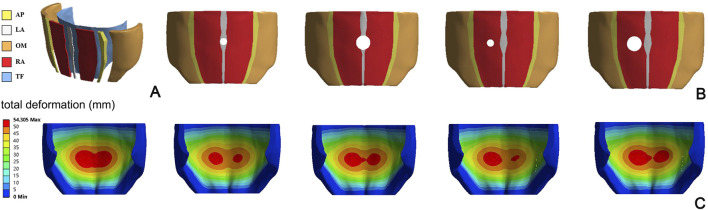
Representation of the FEM model proposed by He et al., 2020 **(A)**, with different locations and size of hernia **(B)**. Magnitude displacement field of the abdominal wall in deformed configuration for different hernia conditions **(C)** Reprinted from He et al. (2020), Copyright 2019, with permission from Elsevier.

Even in the work of He et al. (2020), a numerical model of a human abdomen in passive condition is developed. CT images of a healthy male abdomen are used to reconstruct the geometry of all abdominal structures, including LA, RA, AP, lateral muscles, and TF (Figure 2A). A fiber-reinforced hyperelastic constitutive model is used to describe the mechanical behavior of connective and muscle tissues; constitutive parameters are evaluated based on previous studies (Hernández-Gascón et al., 2013a; Pachera et al., 2016). The focus of this work is to evaluate the effects of surgical repair with meshes of different stiffness on the compliance of the abdominal wall, depending on the position and size of hernia (Figures 2B, C).

**FIGURE 3:**
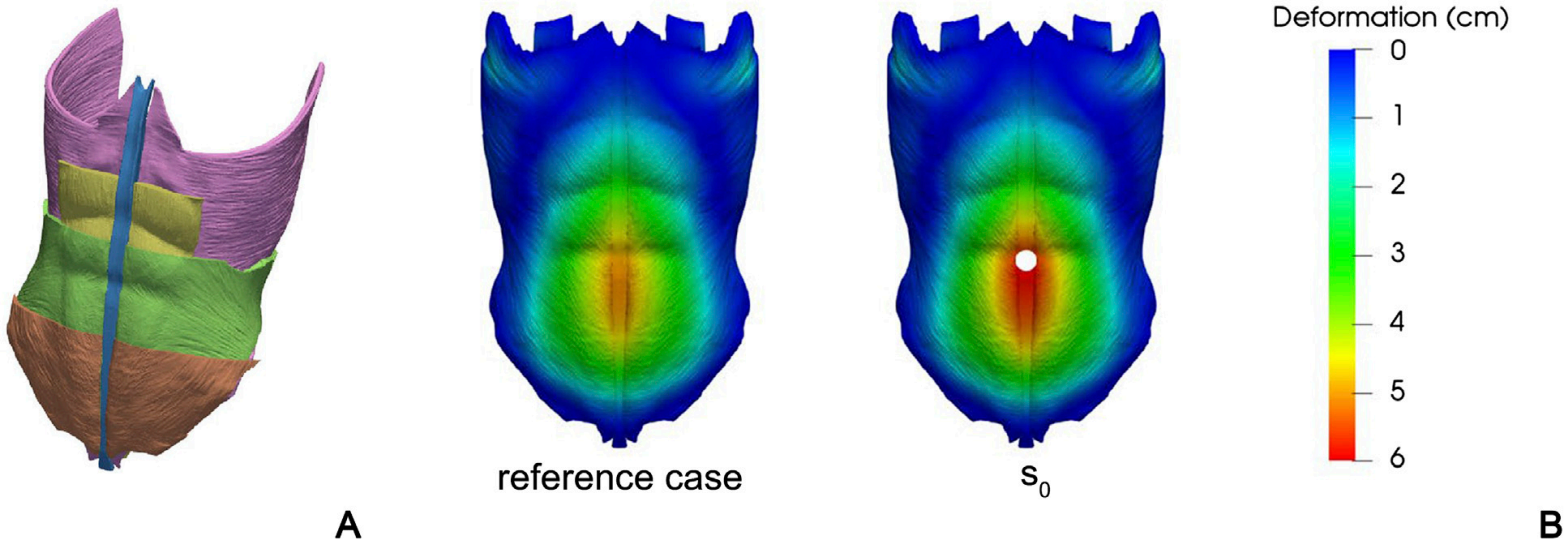
Different anatomical structures of the FEM model developed by Tuset et al., 2022: TA (purple), RA (yellow), IO (green), EO (brown), and LA (blue) **(A)**. Contour of magnitude displacement in a healthy reference case and in a specific stoma position **(B)** This file is licensed under the Creative Commons Attribution 4.0 International license. Figures come from Tuset et al. (2022).

Tuset et al. (2022) develop a FEM model to study the influence of stoma locations on the abdominal wall mechanics. The model is based on CT images taken from an anatomy database and includes LA, RA, TA, IO, and EO (Figure 3A). All tissues are described assuming an isotropic linear elastic behavior, with engineering constants based on previous experimental works (Cardoso, 2012; Cooney et al., 2016). Seventeen different locations of the stoma are taken into account to evaluate the effect of increasing the IAP to about 150 mmHg, in terms of deformation of the region of the abdominal wall next to the stoma. An example of a specific position of the stoma is shown in Figure 3B.

Karrech et al. (2023) develop a FEM model of the abdominal wall aimed at evaluating failure stress around different types of hernia, based on fracture mechanics. The geometry of the model, based on CT data from an anatomy repository, is symmetric with respect to the sagittal plane and encompasses LA, RA, AP, TA, IO, and EO. The hernia is simulated as a damage zone of the abdominal wall in the umbilical position in the LA and RA (incisional hernia). A surgical mesh is included to simulate repair in different surgical conditions (onlay, anterectus, retrorectus, and preperitoneal mesh positioning). The passive response of muscles is modeled assuming them as hyperelastic isotropic materials, with constitutive parameters based on human data (Cardoso, 2012). Surgical mesh is also described as an isotropic elastic material but with linear behavior. The internal surface of the abdominal wall is subjected to an IAP of 6 mmHg, representing the basal IAP in a human subject. The numerical results focus on the evaluation of severe hernia damages and the identification of the best surgical mesh positioning, based on the type and location of hernia.

Since the abdominal wall is largely composed of muscular structures, the effects of muscle contraction on the overall biomechanical behavior must be assessed. In particular, several studies described above take into account high IAP values applied to the abdominal wall in a passive state, while this condition is not true to the physiology of the abdomen.

In this context, the first model capable of describing the active behavior of the abdominal wall is attributed to Grasa et al. (2016), who develop *in vitro* experimental tests and the corresponding FEM models, even tough on a rabbit. The aim of this study is the analysis of the active mechanical response of small rectangular samples taken from the abdominal wall. The numerical models are developed with a geometry resembling the samples and include different layers corresponding to the single abdominal muscles. Constitutive models account for the anisotropic response given by the specific spatial orientation of both collagen and muscle fibers. According to the authors, this constitutive model can be adopted to simulate abdominal biomechanics, extending it to the overall geometry of the wall.

The biomechanical response of the entire abdominal wall of a human subject under muscle contraction is simulated by Pavan et al. (2019). All muscles and fascial tissues are included in the FEM model, considering the specific spatial orientation of the fibers in each muscular layer. Connective tissues are described as fiber-reinforced and almost-incompressible hyperelastic materials, while muscle tissues are modeled with a Hill type three-element formulation (Marcucci et al., 2017). This model can accurately mimic abdominal contraction and assess its deformed shape in relation to IAP corresponding to different daily tasks. The numerical results show a relevant difference in abdominal compliance between passive and active conditions with the same value of IAP.

A refinement of this model is proposed by Todros et al. (2020), adding a structure with a suitable volumetric stiffness, resembling the abdominal cavity. By using this model, it is possible to generate IAP as a direct effect of muscular contraction. This model is partially validated on the basis of experimental data from *in vivo* tests on human subjects.

A similar FEM model is developed by Karami et al. (2023), who refine the constitutive formulation of the muscular tissue considering a chemomechanical approach. In this way, the biomechanical response of the abdominal wall can be related to *in vivo* electromyographic data.

**FIGURE 4:**
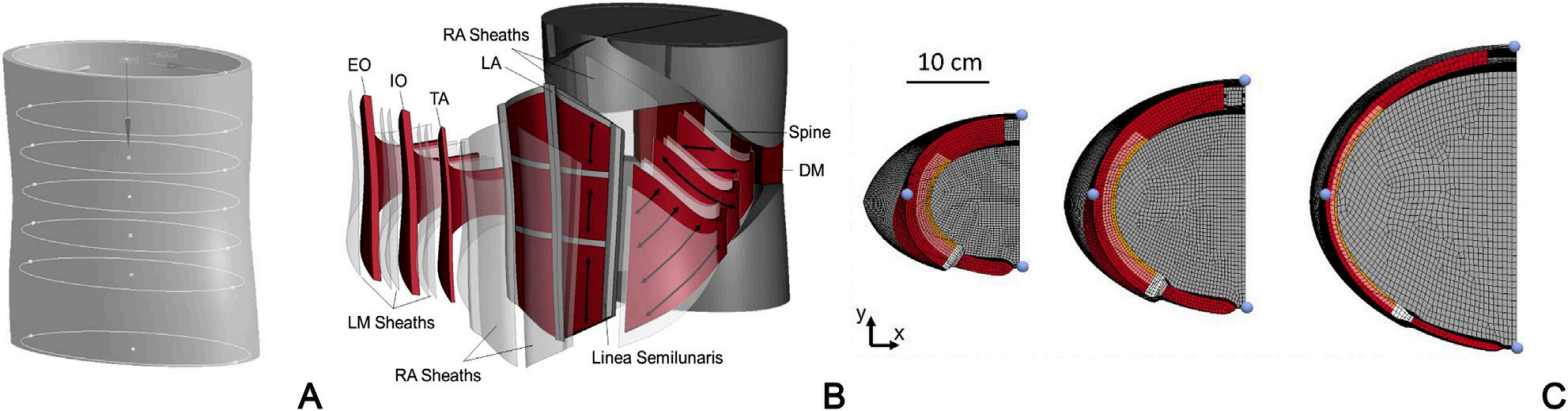
FEM model of the abdominal wall developed by Jourdan et al., 2024: development of the 3D model geometry based on elliptical sections **(A)**; exploded view including all connective and muscular tissues **(B)**; examples of three subject types with different BMI and tissue conformation: normal BMI and high muscular thickness, overweight BMI and medium muscular thickness, obese subjects and low muscular thickness **(C)** Reprinted from Jourdan et al., 2024, Copyright 2023, with permission from Elsevier.

In view of forthcoming patient-specific approaches, Jourdan et al. (2024) develop a FEM model of the abdomen based on a simplified geometry, which is built on seven ellipses placed at different levels along the cranio-caudal axis and scaled to fit the abdomen size of three subject types with different BMI (corresponding to normal, overweight, and obese subjects) (Figure 4). The model includes abdominal muscles (RA, EO, IO, TA and dorsal muscles), bones (ribcage, pelvis, spine), and connective tissue (LA, ARS, PRS, and aponeuroses). As in other previous studies, the connective tissues are modeled as fiber-reinforced almost-incompressible hyperelastic materials and the active behavior of muscles is simulated by means of a Hill type three-element model. This approach aims to analyze the effects of inter-individual variability on the abdominal wall biomechanics in different loading conditions, including both passive (e.g., pre-surgical inflation) and active (i.e., daily motor tasks) behavior.

Technical details regarding all the numerical models previously presented are reported in Table 3. For all the considered articles FEM software is indicated, while other details such as the type of the analyses and the number of degrees of freedom of the models are reported, when available. Table 3 also indicates whether standard constitutive models (at disposal in the software) are adopted for the abdominal wall tissues, or if *ad hoc* constitutive formulations are developed and implemented through specific user subroutines. In spite of the fact that few data about the size of the FEM models are reported in the different studies, it can be assumed that a detailed model of the abdominal wall can be obtained with a number of degrees of freedom in the order of 300,00 ÷ 900,000, largely depending on the extension of the modelled anatomical part. This information can help in estimating the computational complexity associated to a model and in the choice of a computational framework suitable to run these type analyses. Moreover, the non-linearity of the problems related to abdominal wall biomechanics (material non-linearity, large strains, contact conditions) must be considered, since it represents an additional cause of the computational complexity.

**TABLE 3:** Basic data of the abdominal wall FEM models.

Reference	Software	Type of analysis	Degrees of freedom	Constitutive modeling
Hernández-Gascón et al. (2014)	Abaqus	N/A	885,738^a^	standard
Hernández-Gascón et al. (2013a)	Abaqus	static implicit	N/A	standard and user subroutine
Hernández-Gascón et al. (2013b)	Abaqus	static implicit	N/A	standard and user subroutine
Pachera et al. (2016)	Abaqus	N/A	N/A	standard
Todros et al. (2018)	Abaqus	N/A	N/A	standard
He et al. (2020)	Ansys	N/A	N/A	standard
Tuset et al. (2022)	Code_aster	N/A	N/A	standard
Karrech et al. (2023)	Abaqus	N/A	N/A	standard
Grasa et al. (2016)	Abaqus	N/A	N/A	user subroutine
Pavan et al. (2019)	Abaqus	quasi-static implicit	N/A	standard and user subroutine
Todros et al. (2020)	Abaqus	N/A	N/A	standard and user subroutine
Karami et al. (2023)	Abaqus	N/A	N/A	user subroutine
Jourdan et al. (2024)	Ansys	N/A	N/A	standard

^a^ Estimated on the basis of the number of nodes and type of elements.

The literature described above shows that the general trend in this research field is characterized by an increasing refinement of the constitutive models of the abdominal muscles and the addition of the abdominal cavity, which allow for a more realistic description of the abdominal biomechanics. However, the increased model complexity and the multiple interactions among muscular activation, IAP variation, and abdominal wall deformation in different motor tasks require extensive *in vivo* data for model validation.

## 6 *In vivo* characterization of abdominal muscle activation

### 6.1 Electromyography

EMG is a technique commonly used to assess muscle recruitment, measuring myoelectric activity in response to nerve stimulation. The ability of a muscle to respond to this stimulation is evaluated in terms of an action potential signal, whose intensity and shape depend on the number of motor units involved in the task. Applied to the abdominal region, EMG measurements provide data on the excitation pattern of the abdominal muscles during various activities. These data could be an important part of the validation process of numerical models that include the active behavior of the abdominal muscles. A relevant review on studies in the literature investigating EMG measurements during abdominal exercises is proposed by Monfort-Pañego et al. (2009). While reporting more than 80 studies mainly on healthy subjects, the authors highlight that EMG signals are not always normalized to the maximum voluntary contraction, thus limiting the possible comparison between different conditions.

Specific studies take into account different abdominal strengthening exercises that elicit varying levels of RA, IO, and EO excitation (Drysdale et al., 2004; Urquhart et al., 2005; Moraes et al., 2009; Bjerkefors et al., 2010; Escamilla et al., 2010; Okubo et al., 2010; Crommert et al., 2011; García-Vaquero et al., 2012; Czaprowski et al., 2014; Kim S. Y. et al., 2016; Kim et al., 2016 C. R.; Min-Kyu et al., 2016; Southwell et al., 2016; Anders and Steiniger, 2018; Vaičienė et al., 2018; Mandroukas et al., 2022).

Despite the large availability of experimental data, myoelectric activity patterns are rarely used for the development and validation of models of the active muscular behavior. Some studies (Biewener et al., 2014; Knodel et al., 2022; Schwaner et al., 2024) propose different approaches to correlate *in vivo* muscle dynamics based on EMG signals to *in situ* force length and force-velocity functions, aiming at the development of Hill type constitutive models. However, this approach, which could provide an advancement in muscle constitutive modeling, is not applied to the muscles of the human abdominal wall.

### 6.2 Ultrasound imaging

Another non-invasive technique used to quantitatively assess abdominal muscle contraction is US imaging. By providing visual feedback, US images offer valuable information on muscle activation, coordination, and function (ShahAli et al., 2019). The US images are obtained by means of a transducer-equipped ultrasound machine: a high-frequency sound wave emitted by the transducer is used to create a real-time image of the muscles, which is analyzed to evaluate the variation of muscle architecture and thickness during contraction and relaxation (Leighton, 2007).

Different authors evaluate the thickness of abdominal muscles at rest and during contraction in different positions (Hodges et al., 2003; Ainscough-Potts et al., 2006; Norasteh et al., 2007; Brown and McGill, 2008; Mew, 2009; Arab et al., 2013; Nabavi et al., 2014; ShahAli et al., 2015; Tran et al., 2016; Pirri et al., 2019; Johnson et al., 2021; Lin et al., 2021) or during expiratory loading (Kaneko et al., 2006).

Others focus on the difference in myogenic activation between healthy and pathological patients with chronic lower back pain (Vasseljen and Fladmark, 2010; Rasouli et al., 2011; Pulkovski et al., 2012), or on the effectiveness of rehabilitation in strengthening deep abdominal muscles, particularly the TA (McGalliard et al., 2010; Ishida and Watanabe, 2013; Sugimoto et al., 2018; Park et al., 2022). Further studies investigate possible differences in the size and conformation of the abdominal wall due to sex (Rho et al., 2013) or postpartum conditions (Coldron et al., 2008).

### 6.3 Combined EMG and ultrasound measurements

Although EMG and US imaging are effective tools to evaluate different features related to abdominal muscle contraction, each measurement alone is not sufficient to develop and validate FEM models of the abdominal wall. The increase in muscle thickness measured by US imaging during different motor tasks is generally interpreted as an indicator of muscle force generation. However, US measurements should be correlated with EMG data acquired simultaneously on the same subject, to associate muscle thickness increase with a specific myogenic activation.

Few studies consider the coupling of EMG and US to evaluate abdominal muscle contraction, since the correlation between increased muscle thickness and myogenic activation is still controversial. Although some authors try to find a positive correlation between muscle thickness in US images and EMG activity of the abdominal muscles, inconsistent relationships are generally identified during different motor tasks (Hodges et al., 2003; McMeeken et al., 2004; John and Beith, 2007; Brown and McGill, 2010). In addition, some studies (John and Beith, 2007; Coghlan et al., 2008) highlight a decrease in the thickness of lateral abdominal muscles during specific tasks.

Other approaches are proposed to evaluate the anatomical and mechanical properties of muscular motor units through the integration of high-density surface EMG and ultrafast US imaging in other skeletal muscles (Waasdorp et al., 2021; Carbonaro et al., 2023), identifying the regions where single motor unit fibers are located within the muscle cross-section *in vivo*. However, this approach is not directly applicable for the development and validation of FEM models at a larger scale.

### 6.4 Intra-abdominal pressure measurement

The IAP is defined as the pressure within the abdominal cavity that results from the interaction between the abdominal wall and the viscera. The physiological value of IAP oscillates due to the respiratory phases and to the activation of abdominal muscles (Milanesi and Caregnato, 2016).

The value of IAP can be measured directly or indirectly. Direct measurements are obtained by means of a needle or catheter in the peritoneal space, and IAP is measured using a fluid column or pressure transducer system (Risin et al., 2006). This method is generally considered the most accurate, even if it could be associated with side effects such as intestinal perforation and peritonitis. Indirect methods involve the measurement of the pressure transmitted to the lumen of an intra-abdominal structure or organ, including intragastric, intrarectal, intrauterine, intravesical, and vena cava access (Davis et al., 2005). Due to the common practice of intravesical catheterization, IAP is more frequently measured indirectly from intra-bladder pressure (IBP), since Kron et al. (1984) first proposed instrumental measurements with fluid-filled catheters. The reliability of this method is not fully established, and human studies correlating IAP with IBP are limited to few subjects and not entirely reproducible (Malbrain, 2004; Al-Abassi et al., 2018). Nonetheless, this indirect measurement is widely adopted in clinical practice and the IAP value is generally assumed to be equal to the IBP value. A comprehensive review of the different types of IAP sensors and their features, in terms of miniaturization, remote monitoring, and multiplexing is provided by Liao et al. (2021).

Basal IAP is usually less than 7 mmHg in healthy adults, while higher physiological baseline levels (9 to 14 mmHg) are found in morbidly obese patients (De Keulenaer et al., 2009). One of the first comprehensive studies assessing the value of IAP during different motor tasks is carried out by Cobb et al. (2005), who examine a group of 20 subjects (10 male and 10 female) with a mean age of 22.7 years and an average BMI of 24.6. No differences are recorded between men and women; however, the results exhibit high variability between subjects.

Chionh et al. (2006) consider 58 patients (40 men and 18 women) with an age range between 31 and 92 years and measure IAP in supine position and at different degrees of back rising. An increase in the mean IAP value is found as patient position becomes more upright: the mean values of IAP in the supine, 30° and 45° positions are 7.7 mmHg, 9.6 mmHg and 11.0 mmHg for men, and 5.1 mmHg, 7.0 mmHg and 9.6 mmHg, for women. Blazek et al. (2019) review the IAP measurements acquired in several studies during the Valsalva maneuver and different resistance exercises, reaching extremely high values of IAP over 200 mmHg in specific exercises such as squats and deadlift (Kawabata et al., 2010; 2014). Soucasse et al. (2022) propose an extensive analysis of IAP through an intragastric wireless sensor in 20 healthy subjects, both during supervised exercises and during their daily activities. Interestingly, this study highlights that during daily life the IAP values exceeding 50 mmHg, 100 mmHg, and 150 mmHg can be detected on average five times, twice, and once per hour, respectively. Kawabata and Shima, (2023) couple the measurements of IAP, by means of a pressure transducer placed intra-rectally, and EMG on fourteen healthy subjects, to evaluate the combined effect of different breathing patterns and postures on the activation abdominal muscle and the consequent IAP increase. Combining different methods for assessing muscular activity and IAP *in vivo* could be very useful for the development of FEM models of the abdominal wall.

**FIGURE 5:**
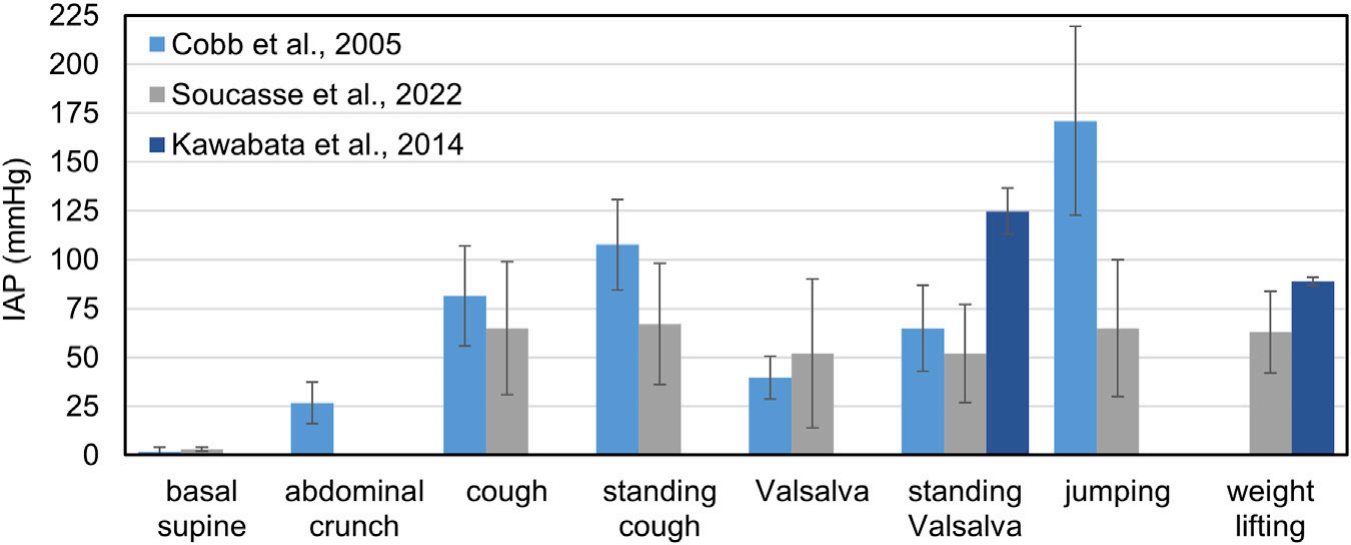
Mean values of IAP measured during several motor tasks by different authors.

A summary of the IAP values measured in different studies for specific motor tasks is reported in Figure 5.

### 6.5 Stiffness measurements

In daily clinical practice, abdominal stiffness is commonly assessed by palpation (Mota et al., 2013), which means that gentle pressure is applied manually to the abdomen and the wall compliance is evaluated qualitatively.

However, more accurate quantitative measurements are needed for the development of FEM models of the abdominal wall. To this purpose, several evaluations are made in the literature to assess the stiffness of the abdominal wall using different techniques, mainly in passive conditions. Some authors measure abdominal wall stiffness during inflation of the abdominal cavity with air. Van Ramshorst et al. (2011) perform a coupled *in vitro-in vivo* study: the abdomen of fourteen cadaveric subjects is insufflated in the *in vitro* study, whereas forty-two healthy subjects are enrolled to perform different motor tasks in the *in vivo* study. Using a custom-made indentation device, the stiffness of the abdominal wall is estimated in different regions (i.e., LA, RA, and lateral muscles), while recording the IAP values corresponding to the increase in the volume of inflation. This study demonstrates a correlation between IAP and the increase in abdominal stiffness, both in passive and active conditions. Tran et al. (2016) evaluate the local stiffness of the abdominal wall in eleven healthy subjects during different motor tasks, using ultrasound shear wave elastography. They show a significant increase of abdominal wall stiffness during muscle activation in the Valsalva maneuver and find that the values of local stiffness are more homogenous on the overall antero-lateral wall during muscular contraction than in passive conditions. Although shear wave elastography is a well-established technique (Dubois et al., 2015), its reliability may be affected by the difficulties in replicating and maintaining voluntary contraction during acquisition.

Remus et al. (2024) analyze the mechanical response of the antero-lateral abdominal wall of ten healthy subjects in different lying positions applying local indentation with a hemispherical probe and monitoring the displacement of the abdominal wall surface through 3D optical measurements. The acquisition is performed during both muscle contraction and relaxation, continuously monitoring myogenic activation with EMG. The force-displacement data obtained are used to estimate the stiffness of the abdominal tissue. Even in this study, an increase in the mean stiffness of the abdominal wall is found during muscle activation, with no significant differences between the regions considered for measurement acquisition. Moreover, inverse FEM modeling is used to estimate the constitutive parameters that allow to simulate the experimental behavior of the abdominal wall tissues. This study shows that local stiffness measurement, coupled with other experimental data such as 3D geometric reconstruction and continuous EMG acquisition on the same subject can be adopted for the development and refinement of FEM models of the abdominal wall.

### 6.6 Surface deformation measurements

Another relevant aspect in the analysis of the abdomen biomechanics is related to the variation in the shape of the abdominal wall due to muscle activation in different motor tasks. While the passive abdomen is uniformly bulging under increasing IAP during inflation, the activation of the abdominal muscles induces specific deformed shapes of the antero-lateral wall depending on the different activation level of each muscle and related trunk motion. Quantitate data on 3D abdomen surface geometry are critically important for the validation of FEM models.

Szymczak et al. (2012) evaluate the abdominal wall deformation of eight healthy subjects during different standing movements, such as bending, stretching, and expiration, through.

The acquisition of markers position on the abdomen surface with two cameras placed in front of the subject. This method allows creating a surface that resembles the subject-specific abdomen in a relaxed standing position and during movements.

Differently, Todros et al. (2019) use laser scanning technique to acquire the surface of the abdominal wall of ten healthy subjects in a relaxed supine condition and during abdominal crunch. Their results show that muscular contraction induces an elevation of the abdominal wall in the region adjacent to LA in the posterior-anterior direction and a concurrent lowering of the lateral muscles in the mediolateral direction. Statistical analyses show a significant difference between the surfaces of the relaxed and contracted abdominal wall for each involved subject. The laser scanning technique adopted in this work is an accurate and reliable method of evaluating surface changes in the abdominal wall during muscular contraction. Nonetheless, as other optical methods of surface analysis, the presence of skin and subcutaneous fat tissue limit the reliability of the investigation to very thin subjects.

**FIGURE 6:**
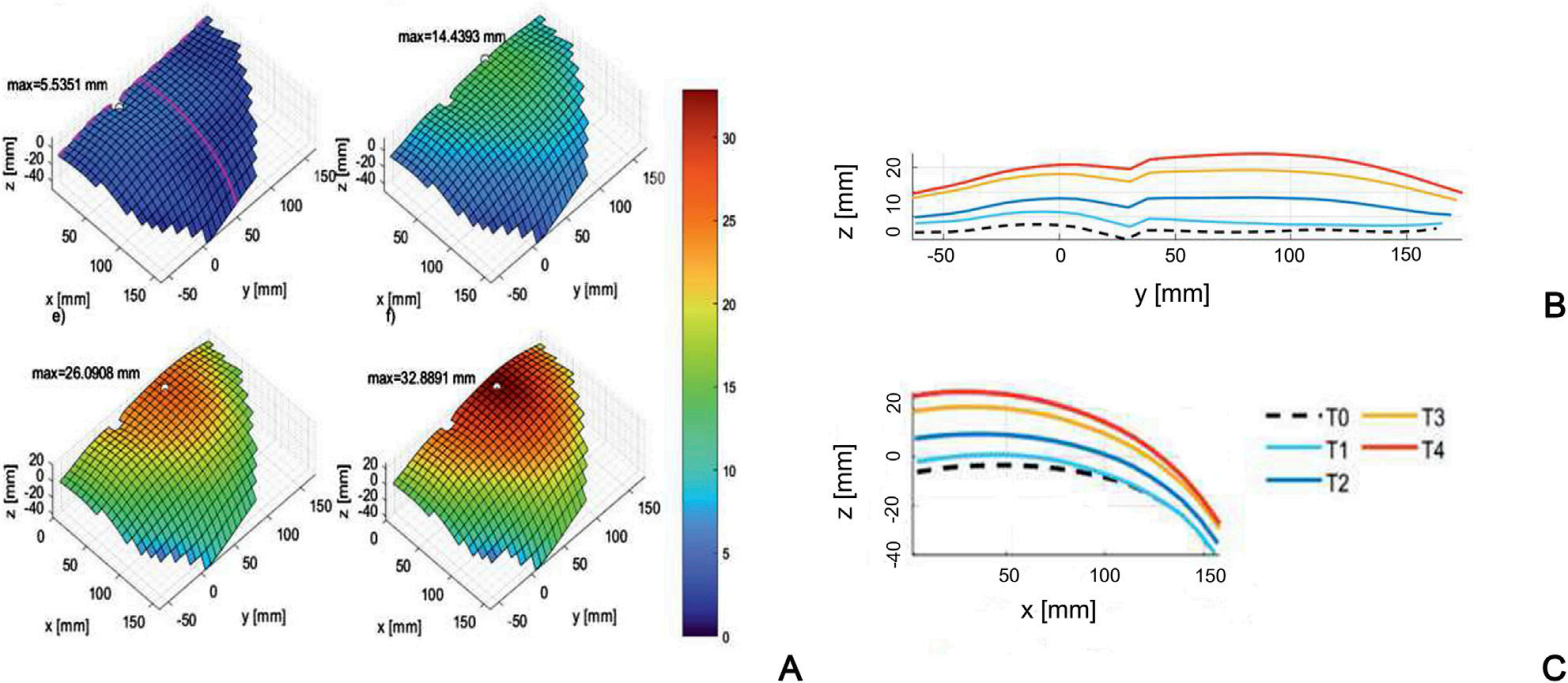
Surface of the abdominal wall of a subject **(A)** at different levels of the inflation process (T0÷T4), with corresponding profiles in craniocaudal **(B)** and mediolateral **(C)** sections. Reprinted from Szepietowska et al. (2023), Copyright 2023, with permission from Elsevier

Lubowiecka et al. (2022) acquire *in vivo* optical measurements to determine the geometry of the abdominal wall while increasing the IAP, and build subject-specific 3D surface geometry of the anterior abdominal wall before and after the increase of IAP. This work provides information on the strain range of the living human abdominal wall in passive condition, showing strain up to 17% at maximum IAP of 13.6 mmHg. However, the investigation is limited to passive behavior and cannot be adopted to validate FEM models that simulate the active behavior. Similarly, Szepietowska et al. (2023) use digital image correlation to evaluate the deformation of the abdominal wall of twelve healthy subjects at different IAP levels (Figure 6). They highlight that the specific abdomen deformations found in each subject are difficult to correlate with IAP, due to a high variability among the mechanical properties of the abdominal tissues of the subjects.

A more accurate way to evaluate the deformation of the abdominal wall is dynamic MRI, which allows for repeated imaging of the trunk region and monitoring of both the superficial and deep components of the abdomen. Jourdan et al. (2021) develop a semi-automatic method for post-processing of dynamic MRI images to quantify the deformations of the abdominal wall muscles in ten healthy subjects during controlled breathing. This approach is then used (Jourdan et al., 2022) to compare the effect of different motor tasks, such as forced breathing, coughing, and Valsalva maneuver, on the abdominal wall geometry of twenty healthy subjects. The results show that in all the exercises lateral muscles shortening, thickening and inward displacement is observed. On the other hand, inhalation is correlated with a large outward displacement of RA muscles.

In general, the combination of different techniques, including surface imaging associated with IAP measurement, or dynamic MRI with related image analysis and post-processing, represent useful tools to evaluate abdominal wall deformation during abdominal contraction and, therefore, to validate FEM models.

## 7 Discussion

As highlighted in the studies presented in this review, FEM-based numerical modeling can potentially be a valuable tool to improve our understanding of abdominal biomechanics in both healthy and pathological states. Computational approach may allow assessing the response of the abdominal wall under different loading conditions, such as under persistently elevated IAP induced in the abdominal compartment syndrome, or post-operative configuration, such as in hernia repair with surgical meshes.

The key aspect in the development and use of numerical models in the biomechanical field, and therefore also in the field of abdominal biomechanics, probably lies in their validation. This is a process which is implemented on experimental data obtained with various techniques. These techniques are used to assess a range of factors, including muscle contraction, muscle thickness, IAP variation, and abdominal surface deformation *in vivo* in human subjects. This is done with the aim of obtaining a comprehensive dataset that can be used to validate these models. Given the considerable complexity and variability of the anatomical site, the validation of a model seems to be intrinsically related to the need to be patient-specific, to consider multiple factors, such as anthropometric dimensions, muscular architecture, biomechanical characteristics of the various tissues, and the stiffness of the abdominal cavity, among others. Although this is undoubtedly the ideal approach, it is important to recognize that many of the techniques used to evaluate the necessary experimental data are, to some extent, invasive. Furthermore, it is understandable that such an approach may not be feasible as a routine one, particularly in view of the associated costs and time constraints. For instance, one might consider the evaluation of intra-abdominal pressure in various subjects as a function of the level of muscle activation or the intrinsic characteristics of muscle fibers, the latter of which can only be obtained through biopsies. It seems that these reasons may be the basis for the fact that the models proposed in the literature thus far have only been partially validated. This is not intended as a criticism of the various approaches, all of which are very rigorous from a scientific standpoint. Rather, it is simply a reflection on the inherent complexity of the problem. For this reason, the scientific community should be encouraged to build a public database containing all the necessary details of experimental protocols and corresponding results.

In the literature, there are both models that focus only on the passive behavior of the abdomen, and models that consider the phenomena of muscle contraction, typical of various motor tasks. Intuitively, models that consider the active behavior of the abdominal muscles appear potentially more effective for describing the behavior both in healthy conditions and in other conditions, for example, after post-surgical repair of abdominal hernias. Models that consider the active behavior of muscles are obviously more complex in their construction, but also require additional efforts for their validation. Integrating measurement of muscle thickness, activation, deformation, and variation in IAP is essential to improve the understanding of abdominal muscle function and its role in various contexts, such as injury prevention and rehabilitation. It should be noted, however, that each different measurement technique alone cannot fully describe the behavior of the abdominal wall structure. To properly assess active muscle behavior, all the available information from different test methods should be combined to better understanding of abdominal biomechanics and improve the accuracy of numerical models.

It seems reasonable to assume that in the future there will be a growing number of patient-specific models of the abdomen, at least in terms of anatomical data. It is already possible to reconstruct anatomical details, such as the thickness of muscles or fascial systems, using highly versatile software. This would require the experimental basis available with MRI, CT, or ultrasound methods. It is also worth noting that there is still room for improvement in terms of the constitutive modeling of the mechanical characteristics of the tissues, whether they are connective or muscular. Obviously, these characteristics cannot be obtained from the subject/patient on which to define the model. It seems reasonable to suggest making every effort to broaden as much as possible the mechanical data on the various tissues from *in vitro* or *ex vitro* tests. This would allow to build a data set that would enable us to define confidence intervals for the various tissues (for example, stiffness for fascial tissues, force-length curves and maximum isometric tension for muscle tissues). These confidence intervals could then be used in the numerical models to obtain the corresponding response intervals from the models.
